# Modulation of striatal glutamatergic, dopaminergic and cholinergic neurotransmission pathways concomitant with motor disturbance in rats with kaolin-induced hydrocephalus

**DOI:** 10.1186/s12987-022-00393-1

**Published:** 2022-11-27

**Authors:** Li-Jin Chen, Jeng-Rung Chen, Guo-Fang Tseng

**Affiliations:** 1 Department of Anatomy, College of Medicine, Tzu Chi University, No. 701, Section 3, Jhongyang Rd., Hualien, 97004 Taiwan; 2 Department of Veterinary Medicine, College of Veterinary Medicine, National Chung-Hsing University, Taichung, Taiwan

**Keywords:** Hydrocephalus, Striatum, Medium spiny neuron, Dendritic spine, Dopamine receptor

## Abstract

**Background:**

Hydrocephalus is characterized by abnormal accumulation of cerebrospinal fluid in the cerebral ventricles and causes motor impairments. The mechanisms underlying the motor changes remain elusive. Enlargement of ventricles compresses the striatum of the basal ganglia, a group of nuclei involved in the subcortical motor circuit. Here, we used a kaolin-injection juvenile rat model to explore the effects of acute and chronic hydrocephalus, 1 and 5 weeks post-treatment, respectively on the three major neurotransmission pathways (glutamatergic, dopaminergic and cholinergic) in the striatum.

**Methods:**

Rats were evaluated for motor impairments. Expressions of presynaptic and postsynaptic protein markers related to the glutamatergic, dopaminergic, and cholinergic connections in the striatum were evaluated. Combined intracellular dye injection and substance P immunohistochemistry were used to distinguish between direct and indirect pathway striatal medium spiny neurons (d and i-MSNs) for the analysis of their dendritic spine density changes.

**Results:**

Hydrocephalic rats showed compromised open-field gait behavior. However, male but not female rats displayed stereotypic movements and compromised rotarod performance. Morphologically, the increase in lateral ventricle sizes was greater in the chronic than acute hydrocephalus conditions. Biochemically, hydrocephalic rats had significantly decreased striatal levels of synaptophysin, vesicular glutamate transporter 1, and glutamatergic postsynaptic density protein 95, suggesting a reduction of corticostriatal excitation. The expression of GluR2/3 was also reduced suggesting glutamate receptor compositional changes. The densities of dendritic spines, morphological correlates of excitatory synaptic foci, on both d and i-MSNs were also reduced. Hydrocephalus altered type 1 (DR1) and 2 (DR2) dopamine receptor expressions without affecting tyrosine hydroxylase level. DR1 was decreased in acute and chronic hydrocephalus, while DR2 only started to decrease later during chronic hydrocephalus. Since dopamine excites d-MSNs through DR1 and inhibits i-MSNs via DR2, our findings suggest that hydrocephalus downregulated the direct basal ganglia neural pathway persistently and disinhibited the indirect pathway late during chronic hydrocephalus. Hydrocephalus also persistently reduced the striatal choline acetyltransferase level, suggesting a reduction of cholinergic modulation.

**Conclusions:**

Hydrocephalus altered striatal glutamatergic, dopaminergic, and cholinergic neurotransmission pathways and tipped the balance between the direct and indirect basal ganglia circuits, which could have contributed to the motor impairments in hydrocephalus.

## Background

Hydrocephalus is a pathological condition characterized by abnormal accumulation of cerebrospinal fluid causing ventricular enlargement in the brain. Enlarged ventricles cause brain damage mediated via factors that include stretching of tissues, ischemia [1] and metabolic disturbance [2]. Hydrocephalus causes apparent movement impairments [3] but the underlying pathophysiology remains largely unexplored.

The basal ganglia consist of a group of subcortical nuclei which are involved in movement control [4]. The striatum, which is the largest basal ganglia structure, receives excitatory glutamatergic projections from both the cerebral cortex and thalamus. Approximately 90% of the striatal neurons are medium spiny neurons (MSNs) with a high density of dendritic spines. Dendritic spines are structures which mediate the vast majority of the excitatory synaptic transmission in brain [5]. MSNs, which use GABA as neurotransmitter, receive cortical and thalamic inputs and give rise to striatal output. Their axons are connected to both direct and indirect basal ganglia pathways. Direct-pathway MSNs (d-MSNs) facilitate movements upon activation of the direct-pathway, while on the other hand, activation of the indirect-pathway via indirect-pathway MSNs (i-MSNs) inhibits movements [6, 7]. In addition, MSNs are also modulated by dopamine released from nigrostriatal terminals and by acetylcholine from cholinergic interneurons within the striatum. Dopamine excites the direct pathway via type 1 dopamine receptors (DR1) expressed on d-MSNs and inhibits the indirect pathway via type 2 dopamine receptors (DR2) expressed on i-MSNs [8]. Acetylcholine, on the other hand, has opposite effects on MSNs, i.e., inhibits the d-MSNs but facilitates the i-MSNs [9]. Equilibration of the actions of these 3 neurotransmitters, namely glutamate, dopamine and acetylcholine, in the striatum is critical to the smooth operation of movements.

The occurrence of movement disorders akin to Parkinson’s disease in hydrocephalic patients has led to the search for cholinergic and dopaminergic changes in the striatum of experimentally induced hydrocephalic rats [10–12]. However, not only is the effect of hydrocephalus on striatal dopamine inconclusive [11, 13], the status of the striatal dopamine receptors is also largely unknown. Results from the few studies exploring the glutamatergic transmission that drives the principal striatal neurons in the hydrocephalic brain have been inconsistent as well [11, 14]. More information on the dendritic spines of d and i-MSNs in hydrocephalus is eagerly awaited as these presumed glutamatergic excitatory foci are prominent features of the cells and are known to be altered in many pathological conditions [15].

In this study, we examined the motor impairments and striatal changes of rats one and five weeks after the induction of hydrocephalus at 3 weeks of age [16] to represent acute and chronic stages, respectively [17, 18]. Changes in the dorsolateral striatum was scrutinized as this area receives mainly sensorimotor cortical projections [19]. Regarding glutamatergic connections, the expressions of the presynaptic markers synaptophysin and vesicular glutamate transporter 1 (VGLUT1), the postsynaptic density protein 95 (PSD95), as well as the expressions of the N-methyl-D-aspartate (NMDA) receptor subunit 1 (NR1) and α-amino-3-hydroxy-5-methyl-4-isoxazolepropionic acid (AMPA) glutamate receptor subunit 1 and 2/3 (GluR1 and GluR2/3) were examined. Dendritic spines, representing excitatory synaptic foci, on d and i-MSNs during acute and chronic hydrocephalus were also quantified. Changes in striatal dopaminergic and cholinergic connections were evaluated by examining the expressions of the respective synthesizing enzymes, tyrosine hydroxylase (TH) and choline acetyltransferase (ChAT). The expressions of DR1 and 2 were also investigated. Together, this study aimed at gaining a better understanding of the pathophysiological changes underlying the motor impairments that occur in hydrocephalus.

## Materials and methods

### Animal preparation and hydrocephalus induction

A total of 125 (85 male and 40 female) 3-week-old Sprague–Dawley rats (45–60 g body weight; Lasco, Ilan, Taiwan) were used in this study. Among these, 9 male rats had severe hydrocephalus with intracranial hemorrhage and died before the end of the experiment, and were thus excluded from all subsequent analyses. In the remaining male rats, 12 were used for in situ whole brain sectioning, 33 for Western blot analysis, 19 for intracellular dye injection, and 12 for immunohistochemistry as described in detail below. Female rats were used only in the initial assessments of behavioral changes. Animals were housed and treated in accordance with the guidelines of the National Laboratory Animal Center and the protocols approved by the Animal Care and Use Committee of the Tzu Chi University (approval number 105081). All efforts were made to minimize animal’s suffering and to reduce the number of animals used.

We followed our earlier protocol to induce hydrocephalus in 3-week-old rats [18]. Briefly, 0.06 ml sterile kaolin suspension (250 mg/ml in 0.9% saline; Sigma-Aldrich, St. Louis, MO, USA) was slowly injected percutaneously into the cisterna magna via a syringe with 26-gauge needle under isoflurane anesthesia (1.5% in oxygen). Rats were allowed to survive for 1 and 5 weeks to represent acute and chronic hydrocephalus, respectively [18]. Age-matched rats injected with an equal amount of 0.9% saline formed the sham-operated, control groups.

Apparent hydrocephalus was consistently induced. The increase in Evan’s ratio, an arbitrary verification of the ventriculomegaly associated with hydrocephalus [20], was verified by calculating the ratio of the greatest width of both lateral ventricles to the greatest width of brain sections at the level of the foramen of Monro.

### Behavioral tests

To evaluate motor ability, we analyzed animals’ open-field activity, motor coordination, and stereotypic movements. Open-field activity tests were conducted in rats 1 and 5 weeks after hydrocephalus induction. Motor coordination and stereotypic movements were assessed a day before the designated acute and chronic hydrocephalus dates, i.e., 6 and 34 days after kaolin injection respectively. Open-field activity, *n* = 8–13 per treatment and/or gender group, was evaluated for arousal, grooming, and gait in a 2-min period. Animal behavior was assessed based on reported criteria [21, 22] and scored with 4 = alert, normal exploration and walking; 3 = slight lethargy and reduced activity but walk normally if prodded; 2 = hunchback position, walk but the gait is waddling or unsteady; 1 = barely able to walk but still feeding; 0 = near death/euthanized. To evaluate motor coordination, an automated rotarod apparatus (3376-4R, Technical and Scientific Equipment, Bad Homburg, Germany) with protocols reported for juvenile hydrocephalic rats [23] was adopted. Male and female rats, 8–13 per group, were studied separately since the performance of male rats was more conspicuously compromised. The test was conducted in two separate trials. First, the endurance was assessed with constant speed, 5 revolutions per minute (rpm) for a maximum of 2 min, and then the ability of the rats to stay on a rotarod accelerating at 0.1 rpm/sec for up to 2 min was assessed. Stereotypic movement was assessed by placing rats individually in a monitor box (Accuscan, Columbus, OH, USA) for 30 min of habituation and then 30 min of behavioral recording. The Accuscan monitor box was made of Plexiglas (42 × 42 × 30 cm) and equipped with infrared beams programmed to detect stereotypic movement. Only the stereotypic movements of the males, 7–9 rats per group, were included in this report since females failed to show any apparent abnormality from their age-matched controls in our preliminary trials.

### In situ whole brain sectioning

To ascertain whether hydrocephalus distorted the striatum, we followed our earlier protocol [17] to prepare whole brain section in situ with skull intact [24]. Three rats per group were studied. Briefly, rats were deeply anesthetized with Zoletil (tiletamine 25 mg/kg and zolazepam 25 mg/kg; Virbac, Carros, France) and xylazine (10 mg/kg; Rompun; Bayer, Leuverkeusen, Germany) intraperitoneally. After decapitation, the whole brain with skull intact was quickly removed and immersed in hexane at − 73 °C. The tissue block was then placed in a stainless-steel container filled with 4–5% carboxymethylcellulose gel and frozen in hexane again. The frozen tissue block was then removed from the container and stored at − 80 °C until sectioning. The tissue block was sectioned serially at 10-μm thickness with a cryostat microtome (CM3050S, Leica, Wetzlar, Germany) using a disposable tungsten carbide blade (Leica). During sectioning, a piece of adhesive film (Cryofilm type IIC, Section-LAB Co., Hiroshima, Japan) was adhered to the cutting surface of the tissue block before preparing each section. After sectioning, the section adhered on one side to the film was removed and treated with 100% ethanol for 10 s, followed by immersion in 4% paraformaldehyde in 0.1 M phosphate buffer (PB) for 1 min to fix the tissue. The tissue section was then stained with hematoxylin and eosin (H&E) and mounted on a glass slide with UV mounting medium (Super cryomounting medium type R2; Section-LAB). After UV irradiation (Section-LAB) to polymerize the mounting medium, the section was ready for light microscopy.

### Western blot analysis

Male rats only were included in this part of the analysis as they exhibited more apparent motor impairments. The rat was sacrificed by decapitation following deep anesthesia as described above. The striatum was quickly removed and the dorsolateral striatum dissected and froze in liquid nitrogen. The brain tissue was then homogenized at 4 °C in tissue protein extraction buffer (#78510) with protease (#78430) and phosphatase (#78420) inhibitors (Pierce; Rockford, IL, USA), and subsequently centrifuged at 10,000*g* for 10 min at 4 °C. The supernatant was collected for further analysis. Protein concentration was determined with BCA protein assay kit (#23225; Pierce). Equal amounts of protein were separated using SDS–polyacrylamide gel electrophoresis and transferred onto a polyvinylidene difluoride membrane (Bio-Rad, Hercules, CA, USA). Primary antibodies employed included mouse anti-synaptophysin (MAB5258; Millipore Billerica, MA, USA), mouse anti-VGLUT1 (MAB5502; Millipore), mouse anti-PSD95 (MAB1598; Millipore), mouse anti-NR1 (32-0500; Thermo-Fisher Scientific, Pittsburgh, PA, USA), rabbit anti-GluR1 (PA1-37776; Thermo-Fisher Scientific), rabbit anti-GluR2/3 (PA1-4660; Thermo-Fisher Scientific), rabbit anti-TH (ab117112; Abcam), rabbit anti-DR1 (ab20066; Abcam), rabbit anti-DR2 (ADR-002; Alomone Labs, Jerusalem, Israel), goat anti-ChAT (AB144P; Millipore) and rabbit anti-glyceraldehyde-3-phosphate dehydrogenase (GAPDH) (sc-25778; Santa Cruz Biotechnology, Santa Cruz, CA, USA) antibodies. Secondary antibodies included goat anti-rabbit (AP132P; Millipore), goat anti-mouse (AP124P; Millipore), and donkey anti-goat (sc-2020; Santa Cruz Biotechnology) conjugated with horseradish peroxidase. Immunolabeled bands were detected with enhanced chemiluminescence reagents (Amersham, Piscataway, NJ, USA). Reactive protein bands were analyzed with Gel-Pro (Media Cybernetics, Silver Spring, MD, USA). The amount of each tissue protein was standardized against that of the GAPDH as the gel-loading control. Uniformity in the expressions of the gel-loading control GAPDH between the control and hydrocephalic groups was verified before analyses (data not shown). The results of the expression of each protein were plotted as a ratio of that of the respective age-matched sham-operated control animals in the histogram of all related figures presented in this study. *N* = 4–12 rats per group for all the analyses in this experiment.

### Intracellular dye injection in combination with substance *P* immunohistochemistry

The dendritic arbor of individual MSN was revealed with intracellular dye injection in brain slices as previously described [18]. Briefly, male rats were deeply anesthetized as described above and transcardially perfused with half-strength fixative, 2% paraformaldehyde, in 0.1 MPB for 30 min. The brain was removed and the area of interest was sectioned immediately into 350-μm-thick coronal brain slices. Slices were then immersed in 0.1 MPB with 10^−7^ M 4′, 6-diamidino-2-phenyl-indole (SI-D9542; Sigma-Aldrich) for 30 min to reveal cell nuclei. A piece of the slices was then placed in a dish covered with a thin layer of 0.1 MPB on the stage of a fixed-stage Zeiss Axioskop microscope equipped with epifluorescence. An intracellular glass micropipette filled with 4% Lucifer yellow (LY, L1177; Thermo-Fisher Scientific) was mounted on a three-axial hydraulic micromanipulator (Narishige, Tokyo, Japan) and visually guided to impale candidate MSN identified by nuclear morphology. The neuron was then filled with LY with negative current generated by an intracellular amplifier (Axoclamp-IIB; Axon Instruments, Foster city, CA, USA). The injected slice was then post-fixed in 4% paraformaldehyde in 0.1 MPB for 3 days and then sectioned into 60-μm-thick serial sections with a cryostat.

To convert the injected LY into a non-fading reaction product and to distinguish between the d and i-MSNs, a double immunostaining technique was employed. Sections from slices with LY-filled neurons were first treated with BLOXALL endogenous peroxidase and alkaline phosphatase blocking solution (SP-6000; Vector Laboratories, Burlingame, CA, USA) for 10 min followed by phosphate-buffered saline (PBS) containing 10% normal goat serum and 0.1% Triton X-100 for 1 h at room temperature. Sections were then incubated with guinea pig anti-substance P (ab10353; Abcam) antibody for 18 h at 4 °C. After several rinses in PBS, sections were incubated with biotinylated goat anti-guinea pig secondary antibody (BA-7000; Vector Laboratories) for 1 h, and then with avidin–biotin-complex alkaline phosphatase conjugate reagent (AK-5000; Vector Laboratories) for another hour at room temperature. The sections were then treated with PBS containing 2% bovine serum albumin and 1% Triton X-100 for 1 h at room temperature. After several rinses in PBS, sections were incubated in biotinylated rabbit anti-LY (A-5751; Invitrogen, Carlsbad, CA, USA) overnight at 4 °C. Sections were then incubated with avidin–biotin-horseradish peroxidase (PK-6100; Vector Laboratories) for 3 h at room temperature. After several rinses in PBS, sections were first reacted with alkaline phosphatase substrate kit (SK-5400; Vector Laboratories) to reveal the substance P labeling. Sections were then washed with 0.05 M Tris buffer (TB; pH 7.4) again before reacted in 0.05% diaminobenzidine tetrahydrochloride (DAB) and 0.01% H_2_O_2_ in TB to reveal the LY-injected neurons. Reacted sections were then mounted on slides, dehydrated, and cover-slipped for observation. Numbers of dendritic spines on the proximal and distal dendrites of both d and i-MSNs were counted with 100X oil immersion objective. Dendritic segments 50–60 and 100–120 μm from the soma were arbitrarily taken as the proximal and distal dendrites of the MSNs, respectively. Numbers of rats, and d and i-MSNs, and their dendritic segments analyzed are listed in Table 1.

**Table 1:** The number of rats, MSNs and dendritic segments of experimental and age-matched control animals analyzed

Experimental groups (number of rats)	MSN types (number of neurons)	Number of dendritic segments	
		Proximal	distal
Acute hydrocephalus (*n* = 4)	d-MSN (*n* = 13)	64	19
	i-MSN (*n* = 23)	184	79
Control: age-matched (*n* = 5)	d-MSN (*n* = 18)	116	47
	i-MSN (*n* = 27)	211	137
Chronic hydrocephalus (*n* = 5)	d-MSN (*n* = 19)	107	59
	i-MSN (*n* = 14)	101	60
Control: age-matched (*n* = 5)	d-MSN (*n* = 22)	130	70
	i-MSN (*n* = 20)	125	86

MSNs, medium spiny neurons; d-MSNs, direct pathway MSNs; i-MSNs, indirect pathway MSNs

### Immunohistochemistry

To investigate whether hydrocephalus altered the localization of TH in the striatum, male rats, 3 per group, were deeply anesthetized as described above and perfused transcardially with 4% paraformaldehyde in 0.1 M PB. The striatum was cryosectioned into 25-μm-thick coronal sections. Sections of interests were first treated with 1% H_2_O_2_ and 0.4% Triton X-100 in 0.1 M PB for 30 min. The sections were subsequently immersed in 10% normal goat serum and 0.4% Triton X-100 in 0.1 M PBS for 1 h, then incubated with rabbit anti-TH antibody (ab117112; Abcam) in 0.1 M PBS overnight at 4 °C. After several rinses in PBS, the sections were incubated with the biotinylated goat anti-rabbit secondary antibody (AP132B; Millipore), and then the standard avidin–biotin-horseradish peroxidase reagents (PK-6100; Vector Laboratories). Sections were then reacted with 0.05% DAB and 0.01% H_2_O_2_ in 0.05 M TB. Reacted sections were mounted on slides, dehydrated, and cover-slipped.

### Statistical tests

All statistical analyses were performed with SigmaPlot 13 (Jandel Scientific, San Rafael, CA, USA). Data presented are mean ± standard error of the mean (SEM). Motor performance data between groups were analyzed with ANOVA on ranks with Dunn’s post hoc test. Dendritic spine densities between groups were tested with unpaired two-tailed Student’s *t*-test when normality was verified, and with Mann–Whitney *U* test if normality failed. Western blot data between groups were analyzed with Mann–Whitney *U* test. Statistical significance was taken at *P* < 0.05.

## Results

In our hands, kaolin injection in juvenile rats consistently induced cerebral ventriculomegaly with an Evan’s ratio close to two-folds of that of the age-matched control rats in a week and increased to more than two-folds of the age-matched control rats 5 weeks post-kaolin injection (Table 2). These two time points were taken as the representative time points for our analyses of the acute and chronic phases of hydrocephalus respectively.

**Table 2:** Comparison of Evan’s ratio of experimental rats versus age-matched control rats

Experimental groups	Evan’s ratio (number of rats)
Acute hydrocephalus	0.36 ± 0.02^*^ (*n* = 4)
Age-matched control of the acute	0.19 ± 0.01 (*n* = 5)
Chronic hydrocephalus	0.45 ± 0.02^*#^ (*n* = 4)
Age-matched control of the chronic	0.19 ± 0.01 (*n = 5*)

^*^P < 0.05 between the marked and its age-matched control rats, ^#^*P* < 0.05 between the marked and the acute hydrocephalic rats

### Hydrocephalus impaired mobility and compressed the striatum

**Fig. 1:**
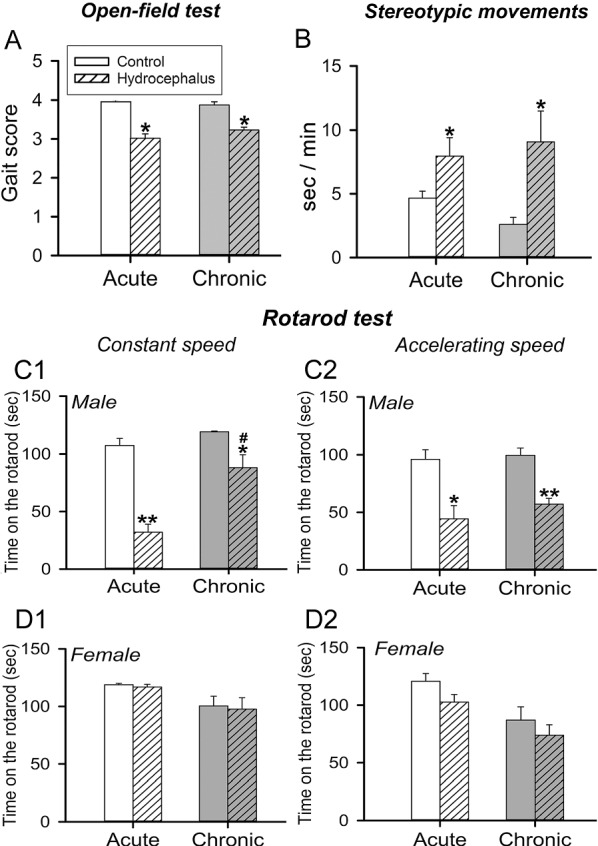
Effects of hydrocephalus on animal behavior and mobility. **A** Bar chart of mean gait scores of the acute and chronic hydrocephalic rats and their corresponding age-matched controls. **B** Bar chart showing the duration of time (s) per minute of male rats that exhibited repetitive/stereotypic behavior. **C1–D2** bar charts of the rotarod performance of the hydrocephalic and age-matched control rats. **C1**, **C2** are bar charts depicting the time (s) male rats stayed on the constant and accelerating-speed rotating rod, respectively while (**D1**) and (**D2**) are plots of the female rats. Mean ± SEM are plotted. *P < 0.05 and **P < 0.001 between the marked and its corresponding age-matched control. ^#^*P* < 0.05 between the marked and the acute hydrocephalic group of the same plot. *n* = 8–13 rats per group

The open-field behavioral analysis showed significantly reduced gait scores (*P* < 0.05) in the hydrocephalic rats (Fig. 1A; gait scores of 3.0 ± 0.1 and 3.2 ± 0.1 for the acute and chronic hydrocephalus versus gait scores of 4.0 ± 0.0 and 3.9 ± 0.1 for the respective age-matched control rats). Besides this, male (Fig. 1B), but not female, hydrocephalic rats, spent significantly more time (*P* < 0.05) displaying stereotypic/repetitive movements than their control counterparts during both the acute and chronic stages. A sex-specific effect was also observed when the rats were challenged with rotarod tests to assess their motor coordination. Hydrocephalus significantly impaired the ability to stay on both constant and accelerating rotarods in male rats (Fig. 1C1 and C2, respectively), but not female rats (Fig. 1D1 and D2). This motor impairment appears to have improved significantly as the hydrocephalus developed from the acute to the chronic phase, as male chronic hydrocephalic rats were able to stay longer on the constant-speed rotarod than the acute counterparts (Fig. 1C1, #*P* < 0.05). The results support the proposition that hydrocephalus impaired motor functions, especially those of the male rats.

**Fig. 2:**
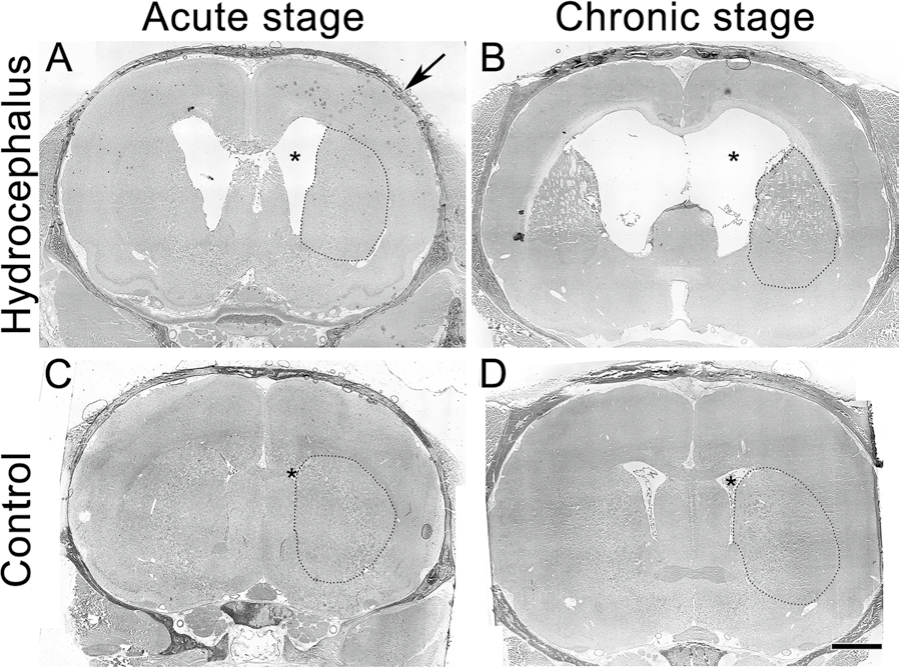
Coronal sections of the whole brain with skull intact from hydrocephalic and age-matched control rats. The brain in situ with skull (arrow) intact was freshly frozen and sectioned coronally at 10-μm thickness. Low-power micrograph of representative H&E-stained sections of the hydrocephalic (upper row) and the age-matched control brains (lower row) are illustrated. **A**, **B** are from acute and chronic hydrocephalic rat, respectively. **C**, **D** are from corresponding age-matched control rats. The striatum was outlined with fine dotted line. Asterisks mark lateral ventricles. Scale bar = 2 mm in **A**–**D**

The brain harvested and sectioned with skull intact, i.e., closely resembling the in vivo state, shows that kaolin injection caused enlargement of the lateral ventricles (asterisks in Fig. 2A, B). The lateral ventricles were larger in the chronic than the acute hydrocephalus, suggesting a progressive enlargement of ventricles over time. The striatum was apparently pushed laterally by the enlarged ventricles (compared the position of the dotted line enclosed areas in Fig. 2A, B with those of the age-matched control rats, Fig. 2C, D respectively), suggesting that hydrocephalus could have affected striatal function.

### Hydrocephalus reduced striatal glutamatergic presynaptic and postsynaptic marker proteins

**Fig. 3:**
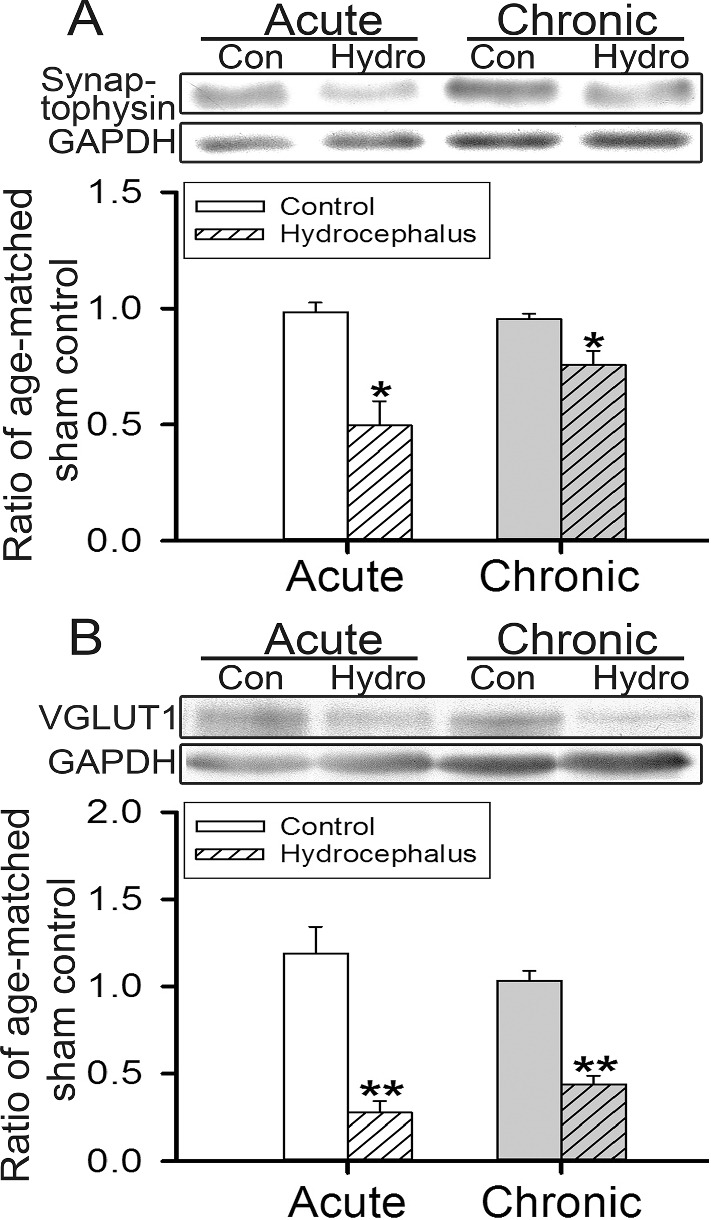
Western blot analyses of synaptophysin and VGLUT1 expressions in the striatum. **A**, **B** are synaptophysin and VGLUT1 respectively. In each panel, representative immunoblots are shown at the top while the histograms of the corresponding analyses at the bottom. The expression of the GAPDH is the internal gel-loading control. Expression of each protein was normalized to that of the GAPDH and presented as a ratio to that of the age-matched sham-operated control animals. Con, control; Hydro, hydrocephalus; VGLUT1, vesicular glutamate transporter 1; GAPDH, glyceraldehyde-3-phosphate dehydrogenase. *P < 0.05 and **P < 0.001 between the marked and its age-matched controls, *n* = 5–10 rats per group

**Fig. 4:**
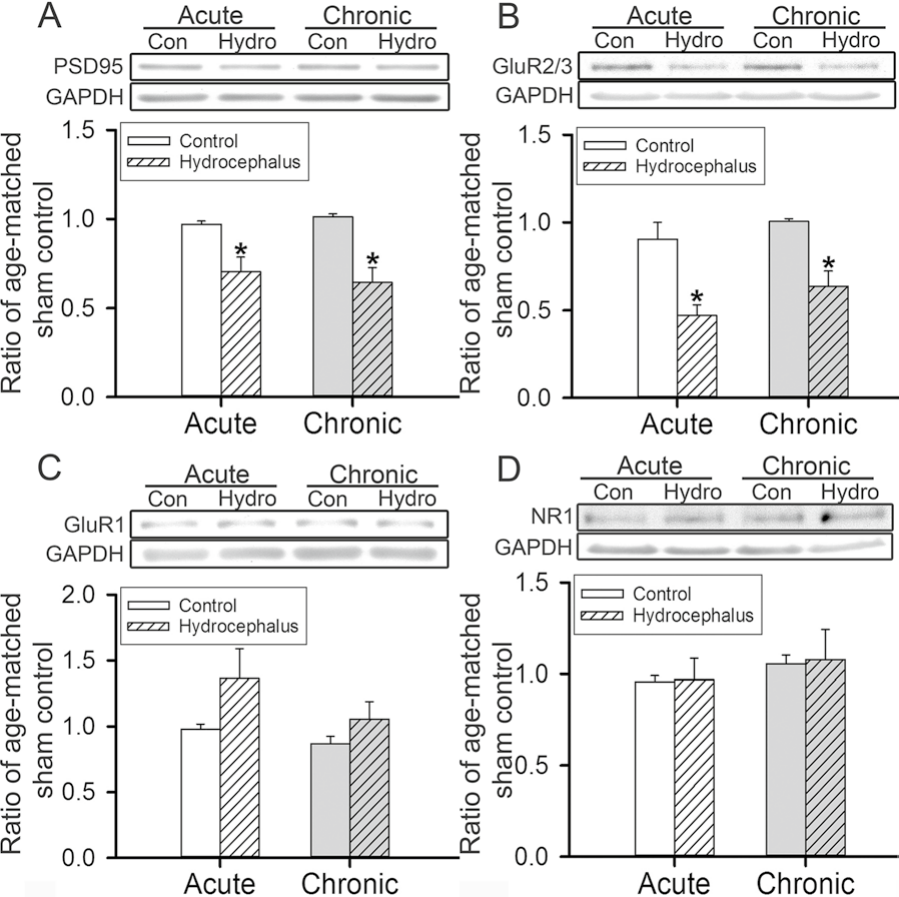
Expression of striatal glutamatergic postsynaptic density protein and receptor subunits. **A**–**D** are the data of PSD95, GluR2/3, GluR1, and NR1 respectively. Representative immunoblots are displayed at the top of each panel with the histograms of corresponding analyses at the bottom. The expression of the GAPDH is the internal gel-loading control. The expression of each protein was normalized to that of the GAPDH and presented as a ratio to that of the age-matched sham-operated control animals. Con, control; Hydro, hydrocephalus; PSD95, postsynaptic density protein 95; GluR2/3 and GluR1, AMPA glutamate receptor subunit 2/3 and 1; NR1, NMDA receptor subunit 1; GAPDH, glyceraldehyde-3-phosphate dehydrogenase. *P < 0.05 between the marked and the corresponding age-matched control, *n* = 4–11 rats per group

Behavioral impairments suggest that hydrocephalus could have altered striatal neural connections to affect function. Western blot analyses show that hydrocephalus, both acute and chronic, significantly reduced the expressions of the presynaptic marker protein synaptophysin and the glutamatergic presynaptic marker VGLUT1 in the striatum (Fig. 3; *n* = 5–10). The expression of the postsynaptic glutamatergic synaptic density protein PSD95 was reduced simultaneously (Fig. 4A; *n* = 5–11). This was accompanied by a reduction of the AMPA receptor subunit GluR2/3, but not GluR1 (Fig. 4B, C, respectively; *n* = 4–12). However, the expression of the NMDA receptor subunit NR1 was not altered (Fig. 4D). These findings support the notion that hydrocephalus could have altered the glutamatergic excitatory neural connections in the striatum.

### Hydrocephalus trimmed dendritic spines on MSNs

**Fig. 5:**
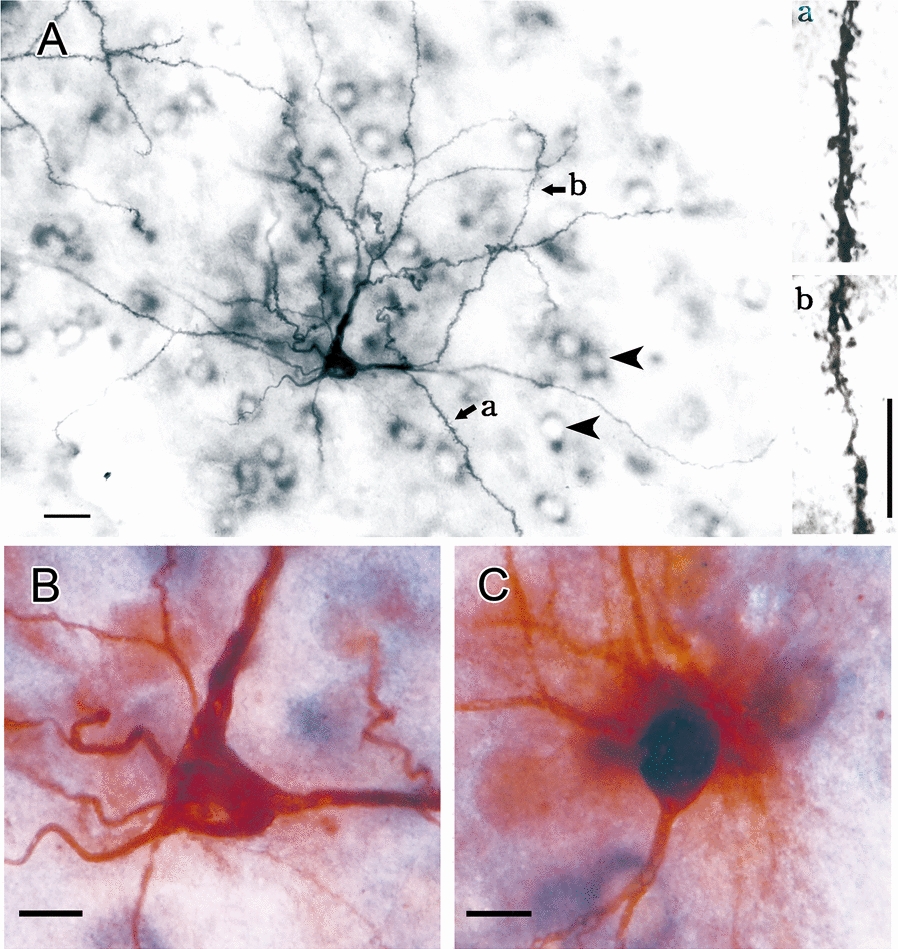
Intracellular dye-injection and substance P immunolabeling of MSNs. **A** A representative LY-filled MSN in a 60-μm-thick section of the injected brain slice from an age-matched control rat of the acute hydrocephalus. The section was also reacted immunohistochemically for substance P. This intracellular dye-filled neuron had a typical MSN morphology. Arrowheads show examples of the substance P-labeled MSN cell bodies without intracellular dye injection in the same section. Substance P-immunoreaction product is in the perinuclear cytoplasm. Insets (*a*) and (*b*) show representative magnified views of a proximal and distal dendritic segment of the LY-filled MSN in (**A**). **B** High-power view of the cell body of the LY-filled MSN in A shows the absence of perinuclear substance-P immunoreaction product. **C** A LY-filled MSN concurrently immunolabeled with substance P. Notice the dark perinuclear immunoreaction product. Scale bar = 20 μm in (**A**) and 10 μm for (**B**, **C**) and insets (*a*) and (*b*)

To explore the morphological correlates of the excitatory connectivity changes, we performed intracellular dye injection of striatal neurons in brain slices prepared from both hydrocephalic and age-matched control rats. Sections prepared from the brain slices containing intracellular dye-filled cells were also reacted with substance P immunohistochemistry to distinguish between d and i-MSNs for only the former express substance P. Figure 5A shows a representative dye-filled MSN in the dorsolateral striatum of an age-matched control rat of the acute hydrocephalus. The micrograph of the cell was taken from a 60-μm-thick section of an injected brain slice after substance P immunohistochemistry. The cell had a stellate morphology with dendrites radiating from the cell body and studded with high densities of dendritic spines (Fig. 5A, insets a and b representing a typical proximal and distal dendritic segment, respectively), typical of the MSN. The cell body of this intracellular dye-filled MSN contained no substance P-immunoreaction product as shown with higher magnification in Fig. 5B. Figure 5C shows a higher magnification of the cell body of a typical substance P-immunoreactive and intracellular dye-filled MSN. The cell body contained dark dense immunoreaction product. Arrowheads in the low-power micrograph in Fig. 5A point to examples of non-intracellular dye-filled, substance P-immunoreactive MSNs.

**Fig. 6:**
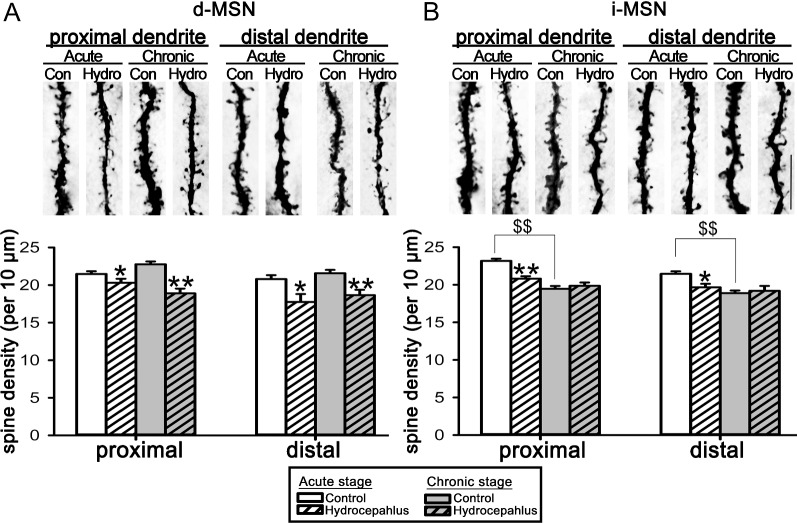
Dendritic spine density analyses of the intracellular dye-filled d and i-MSNs. **A**, **B** are analyses of the d and i-MSNs respectively. The tissue was also processed with substance P immunohistochemistry to distinguish between d and i-MSNs. In each panel, representative proximal and distal dendritic segments of the intracellular dye-filled neurons are shown at the top while the histogram of the corresponding analysis at the bottom. Numbers of dendritic segments, neurons, and rats studied in this part of the experiments are in Table 1. Con, control; Hydro, hydrocephalus; d-MSN, direct-pathway medium spiny neuron; i-MSN, indirect-pathway medium spiny neuron. *P < 0.05 and **P < 0.001 between the marked and the corresponding age-matched control. ^$$^*P* < 0.001 between the age-matched control rats of the chronic and acute groups. Scale bar = 10 μm

To explore whether hydrocephalus affected dendritic spines, spine densities on the representative proximal and distal dendritic segments of the intracellular dye-filled d and i-MSNs were analyzed (Fig. 6; Table 1). The upper part of Fig. 6 shows representative dendritic segments. During the acute hydrocephalus, dendritic spine densities on the proximal and distal dendrites of the d-MSNs were reduced to 83.0–94.5% of those of the age-matched control rats (Fig. 6A), and maintained at this level in the chronic hydrocephalus. On the other hand, the reduction of the dendritic spines on i-MSNs during the acute hydrocephalus had disappeared by chronic hydrocephalus; there were no difference in dendritic spine densities between the experimental and the age-matched control rats during the chronic hydrocephalus (Fig. 6B). Thus, there was a differential effect of hydrocephalus on the densities of the presumed excitatory synaptic foci on the d and i-MSNs, indicating that hydrocephalus might affect the direct and indirect basal ganglia pathways differently. Interestingly, the densities of the dendritic spines on both the proximal and distal dendrites of the control i-MSNs, at 8 weeks of age was significantly lower than those at 4 weeks of age, suggesting a pruning of dendritic spines during this period of the animals’ life.

### Hydrocephalus reduced striatal dopamine receptors and choline acetyltransferase

**Fig. 7:**
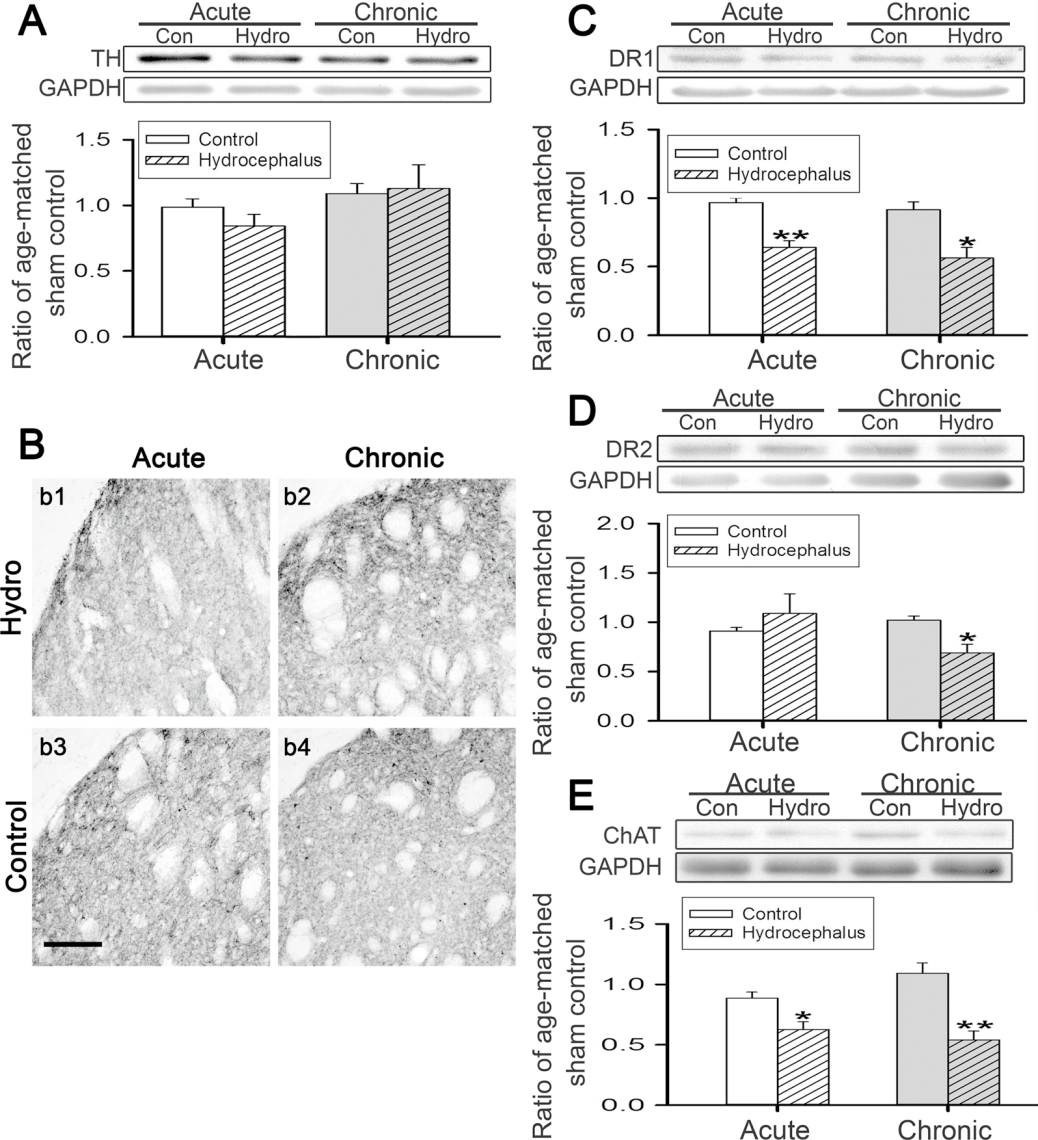
Changes in the expressions of the striatal dopaminergic and cholinergic neurotransmitter markers in hydrocephalic rats. **A** shows representative Western blots of the expression of TH in the striatum of hydrocephalic and age-matched control (*n* = 4–12 rats per group). **B** Representative TH immunolabeling from the dorsolateral striatum of the acute and chronic hydrocephalic (*b1* and *b2*, respectively) and corresponding age-matched controls (*b3* and *b4*) (*n* = 3 rats for each group). **C**–**E** are representative Western blots and corresponding analyses for the expressions of striatal DR1 and 2 and ChAT (*n* = 4–11 rats per group). The expression of the GAPDH is the internal gel-loading control. The expression of each protein was normalized to that GAPDH and presented as a ratio to that of the age-matched sham-operated control animals. Con, control; Hydro, hydrocephalus; TH, tyrosine hydroxylase; DR1 and DR2, type 1 and type 2 dopamine receptors; ChAT, choline acetyltransferase; GAPDH, glyceraldehyde-3-phosphate dehydrogenase. *P < 0.05 and **P < 0.001 between the marked and the corresponding age-matched control. Scale bar = 100 μm

We then evaluated the effects of hydrocephalus on the expressions of the striatal dopaminergic and cholinergic marker proteins since both connections are known to modulate d and i-MSN activities. Both Western blotting (Fig. 7A) and immunohistochemical staining (Fig. 7B) show that hydrocephalus did not alter the striatal TH expression. On the postsynaptic side, the expression of DR1 was reduced during both the acute and chronic hydrocephalus (Fig. 7C), while that of the DR2 was reduced only during the chronic hydrocephalus (Fig. 7D). These suggest that hydrocephalus might have also affected basal ganglia activity by reducing the action of dopamine on the direct pathway earlier than the indirect pathway since d and i-MSNs express principally DR1 and DR2 respectively.

With regard to the striatal cholinergic connection, Western blotting shows the reduced expression of ChAT, the rate-limiting enzyme in acetylcholine synthesis, in acute hydrocephalus and the reduction persisted into chronic hydrocephalus (Fig. 7E). These findings suggest that hydrocephalus might also have an immediate and persistent negative effect on the cholinergic action within the striatum.

## Discussion

**Fig. 8:**
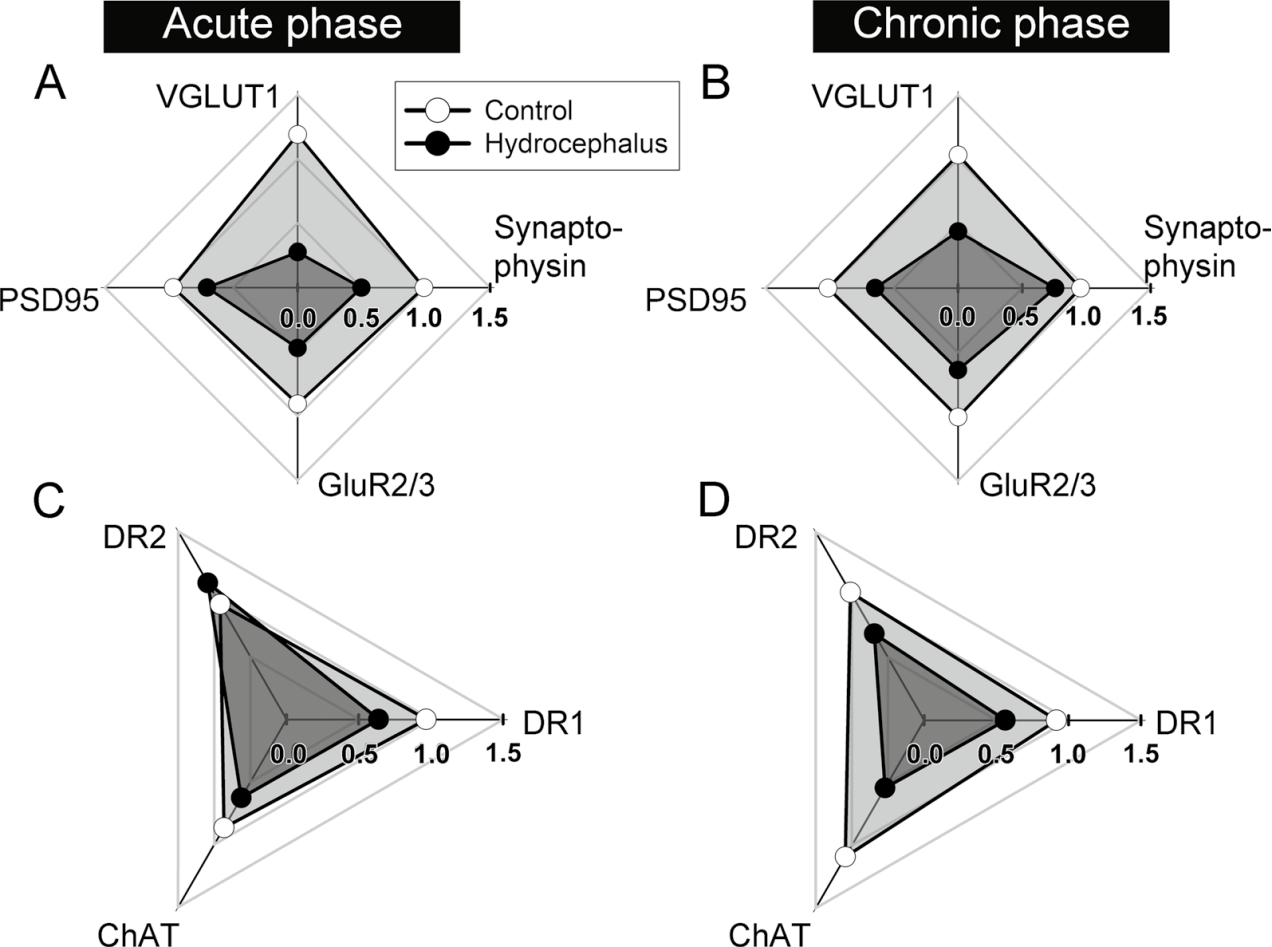
Radar plots summarizing the effects of hydrocephalus on the striatal marker proteins that were investigated. Two-dimensional radar plots was used to depict the expressions of multiple proteins in acute and chronic hydrocephalus in reference to the expressions of their age-matched sham-operated control counterparts. Each axis shows the mean of the hydrocephalic (filled circle) and the corresponding control (empty circle) with 0 expression at the center and full expression out to the periphery. For each data point, the expression of the named marker protein was first standardized to that of the corresponding internal gel-loading control and then to the mean of the age-matched sham-operated group. The area enclosed by the lines connecting between the hydrocephalic or control data points of the adjacent axes represents the relative magnitudes of the expressions of the proteins of the hydrocephalic and age-matched sham-operated control rats respectively. The area enclosed by the data points of hydrocephalic group was shaded darker for easier visualization. Glutamatergic-related protein expressions are grouped and plotted in **A** and **B**, while dopaminergic and cholinergic modulatory action-related are shown in **C** and **D**. The expressions under acute and chronic hydrocephalus were plotted separately as indicated. Hydrocephalus affected glutamatergic connections instantly at the acute stage and persisted to the chronic phase. The effect on DR2 expression was however delayed and showed up during chronic hydrocephalus. VGLUT1, vesicular glutamate transporter 1; PSD95, postsynaptic density protein 95; GluR2/3, AMPA glutamate receptor subunit 2/3; DR1 and DR2, type 1 and type 2 dopamine receptors; ChAT, choline acetyltransferase

The main findings of the present study are that hydrocephalus compromised the mobility of rats and at the same time altered the 3 major striatal neurotransmitter connections. Alterations to the marker protein expressions in these 3 striatal neurotransmitter connections are summarized in Fig. 8 into those affecting the glutamatergic connection (A and B), and those of the modulatory pathways i.e., dopaminergic and cholinergic (C and D). These alterations could have underlain the mobility changes of the hydrocephalic rats.

### Hydrocephalus affected the mobility of male and female rats differently

Behaviorally, hydrocephalic rats showed poorer gait scores (Fig. 1A) which was similar to that reported earlier [21–23, 25, 26]. However, there was no difference in rotarod performance between the hydrocephalic and age-matched sham-operated control animals when male and female rats were combined. To our surprises, male, but not female, rats show poorer rotarod performance when they were analyzed separately (Fig. 1C1–D2). This indicates that there was a sex-specific effect of hydrocephalus on motor coordination and agility. Although we did not find any corresponding sex-specific alteration of striatal neural connection marker proteins, it is interesting to note that the female sex hormone estradiol has been reported to protect dopaminergic neurons and enhance dopaminergic activity in the striatum [27, 28]. This female-specific protective effect may play a role in counteracting the negative effects of hydrocephalus on striatal dopaminergic neural connection, hence maintaining the female rats’ performance in the more demanding motor coordination rotarod test to a level comparable to that of the age-matched sham-operated control rats. Interestingly, Hetherington and Dennis (1999) reported that girls with hydrocephalus performed better than boys on a test of limb ataxia and suggested that sex difference exists in motor performance in hydrocephalus [29]. A definitive sex-dependent difference in the effects of hydrocephalus between the male and female remains to be clarified [30, 31].

### Functional significance of the neurotransmitter system changes in hydrocephalus

Hydrocephalus could immediately affect the striatum which is sited right next to the greatly enlarged lateral ventricles (Fig. 2). Although the striatal neuronal density has been observed to be preserved in hydrocephalic rats [12], the mean striatal blood flow was significantly reduced [1]. This is expected to affect the functions of MSNs since reduction in blood flow impairs neuronal functions [32]. On the other hand, cortical pyramidal neurons were found to have trimmed their dendritic spines under experimental hydrocephalus [18, 33–35], hence were expected to have reduced outputs. With this, corticostriatal influences are likely to be reduced under hydrocephalus since a large part of the excitatory projection to the striatum arises from the cerebral cortex. Consistent with this, we observed a reduction of the general presynaptic marker synaptophysin and the cerebral glutamatergic presynaptic marker VGLUT1 [36] in the striatum (Figs. 3, 8), consistent with a decrease of cortical excitatory influence to the striatum. In line with this, the glutamatergic postsynaptic marker PSD95 in the striatum was also reduced (Fig. 4A), accompanied by a reduction of dendritic spines, the morphological correlates of glutamatergic excitatory synaptic foci, on both the d and i-MSNs (Fig. 6) starting from acute hydrocephalus. Thus, hydrocephalus is likely to have reduced the cortical excitatory inputs to both the direct and indirect basal ganglia pathways. However, the effect on i-MSNs appears to be transient as dendritic spines on i-MSNs during chronic hydrocephalus were comparable to those of the age-matched controls (Fig. 6B).

Glutamatergic receptor-wise, striatal GluR2/3, but not GluR1 or NR1, subunit was significantly reduced. The reduction of striatal GluR2/3 could reflect changes of the MSN’s AMPA receptor subunit composition since in the striatum GluR2 is most common in MSNs rather than interneurons [37]. It’s known that AMPA receptors lacking GluR2 are permeable to Ca^2+^, hence hydrocephalus could have resulted in glutamate toxicity to MSNs due to an increase of Ca^2+^ influx [38]. These imply that hydrocephalus affected striatal functions not only by reducing the cortical excitatory inputs but also by increasing glutamatergic excitotoxicity to the MSNs.

The catecholamine system in the striatum has been the focus of experimental hydrocephalus researches as well [39, 40]. Studies exploring its effects however, have failed to reach a consensus [11, 13]. In the present study, striatal TH level was not affected by hydrocephalus (Fig. 7A, B), in accord with earlier reports that striatal dopamine level remained steady in experimental hydrocephalic rats and rabbits [11, 39, 41]. In the striatum, dopamine binds to DR1 and DR2, expressed preferentially on d and i-MSNs respectively. DR1 activation increases d-MSNs’ excitability [42, 43]. Thus, the persistent decrease of striatal DR1 from acute to chronic hydrocephalus (Figs. 7C, 8) is expected to persistently reduce d-MSN’s excitability, hence downregulating the influence of the direct basal ganglia pathway. On the contrary, dopamine binds to DR2 to inhibit i-MSNs [44]. The late decrease of striatal DR2 during the chronic hydrocephalus (Figs. 7D, 8) suggests a late disinhibition of i-MSNs in the hydrocephalus state. Interestingly, i-MSNs laterally inhibit nearby d-MSNs through GABAergic axon collaterals [45]. This late increase in i-MSN’s activity may additionally suppress d-MSNs at the same time. Thus, there appears to be an added tier of suppression on the direct basal ganglia pathway coming indirectly from the i-MSNs of the indirect pathway as hydrocephalus progresses to the chronic stage. Since in the basal ganglia, the overall action of the direct pathway is to facilitate movement [46], our results are consistent with a negative impact of hydrocephalus on mobility with a more serious motor outcome in chronic hydrocephalus.

Regarding cholinergic connection, acetylcholine enhances i-MSN’s but reduces d-MSN’s excitability [47]. Our finding of a decrease of ChAT expression from acute to chronic hydrocephalus (Figs. 7E, 8) is consistent with an earlier finding that striatal ChAT immunoreactivity in rats was reduced 2 weeks after kaolin injection [12]. Reduction of ChAT could lead to reduced synaptic acetylcholine release, and tip the action of striatal cholinergic circuit to favor the excitation of d-MSNs and the inhibition of i-MSNs at the same time.

### Correlation between neurotransmitter marker changes and behavioral alterations under hydrocephalus

Although the functional relevance of the d and i-MSNs’ activities in movement and control is still far from been clear [48], activation of the d-MSNs is known to promote movements, whereas activation of the i-MSNs inhibits movements. Sippy et al. [46] demonstrated that d-MSNs, but not i-MSNs, contribute to movement initiation in the context of motivation. The hydrocephalus state has apparently shifted the striatal neuronal circuitry to discourage movement as animals showed poorer gait score and rotarod performance than their age-matched control counterparts (Fig. 1). Decrease in the striatal acetylcholine release [49] and the indirect basal ganglia pathway activity [50, 51] have been reported to increase stereotypic movements. This is in agreement with the present findings that hydrocephalus increased the stereotypic movements of rats (Fig. 1B). In fact, a balanced activation of the d and i-MSNs in association with properly-tuned dopaminergic and cholinergic modulatory actions on them are critical to action selection and initiation [52]. In short, our biochemical and morphological findings appear to fit the hydrocephalic animals’ behavioral manifestations. Thus, hydrocephalus could have resulted in striatal neural connectivity changes to affect animal’s behavior.

### Limitations of the present study

Regarding the induction of hydrocephalus with kaolin injection, the injection is known to induce variable degrees of ventricular dilation in rats [53]. This study however did not cover the whole spectrum of the ventricular enlargements. We excluded animals that failed to survive till the experiment’s end point or those with intracranial hemorrhage, both of which were more likely to be severe hydrocephalic cases. The ventriculomegaly in our animals (Table 2) corresponds to the moderate degree hydrocephalus of an earlier classification criteria [53]. In addition, we did not study the relationship between the degree of the ventricular dilation of both the lateral and third ventricles with the extent of the striatal and behavioral changes. Further studies are required to clarify these issues. On the timing of the induction of hydrocephalus in rats, in this study kaolin was injected at 3 weeks of age when the brain was still maturing. Injection of kaolin earlier, at 1 day of age [35], but not at 3 weeks of age [18], was found to induce frequent dendritic varicosities in rat cortical neurons. This suggests that age is a factor affecting the degree of neuronal changes or damages following hydrocephalus. Whether the present findings are comparable to those of the hydrocephalus in adult animals remains to be explored. Lastly, this study explored mainly changes of the striatal neurotransmission-related marker proteins and the dendritic spine densities on MSNs following hydrocephalus. With these, we extrapolated that striatal connectivities and functions might have altered following hydrocephalus. Future studies aiming at directly assessing the striatal neuronal activity in the hydrocephalus condition are warranted to explore the functional meanings of the present findings.

## Data Availability

The dataset used and/or analyzed during the current study are available from the corresponding author on reasonable request.

## Abbreviations

AMPA: α-Amino-3-hydroxy-5-methyl-4-isoxazolepropionic acid
ChAT: Choline acetyltransferase
DAB: Diaminobenzidine tetrahydrochloride
DR1: Type 1 dopamine receptors
DR2: Type 2 dopamine receptors
GAPDH: Glyceraldehyde-3-phosphate dehydrogenase
GluR1: α-Amino-3-hydroxy-5-methyl-4-isoxazolepropionic acid glutamate receptor subunit 1
GluR2/3: α-Amino-3-hydroxy-5-methyl-4-isoxazolepropionic acid glutamate receptor subunit 2/3
H&E: Hematoxylin and eosin
LY: Lucifer yellow
MSN: Medium spiny neurons
d-MSNs: Direct-pathway medium spiny neurons
i-MSNs: Indirect-pathway medium spiny neurons
NMDA: N-methyl-D-aspartate
NR1: N-methyl-D-aspartate receptor subunit 1
PSD95: Postsynaptic density protein 95
PB: Phosphate buffer
PBS: Phosphate-buffered saline
TB: Tris buffer
TH: Tyrosine hydroxylase
VGLUT1: Vesicular glutamate transporter 1
